# Supplementary material for: The causal relationship between the human gut microbiota and pyogenic arthritis: a Mendelian randomization study
Source: Front Cell Infect Microbiol. 2024 Nov 26;14:1452480. doi: 10.3389/fcimb.2024.1452480 (PMC11629706; doi:10.3389/fcimb.2024.1452480)
Supplement: Supplementary file 1 [file DataSheet1.zip › mendelian test/ebi-a-GCST90027765.csv_leave_one_out.pdf]

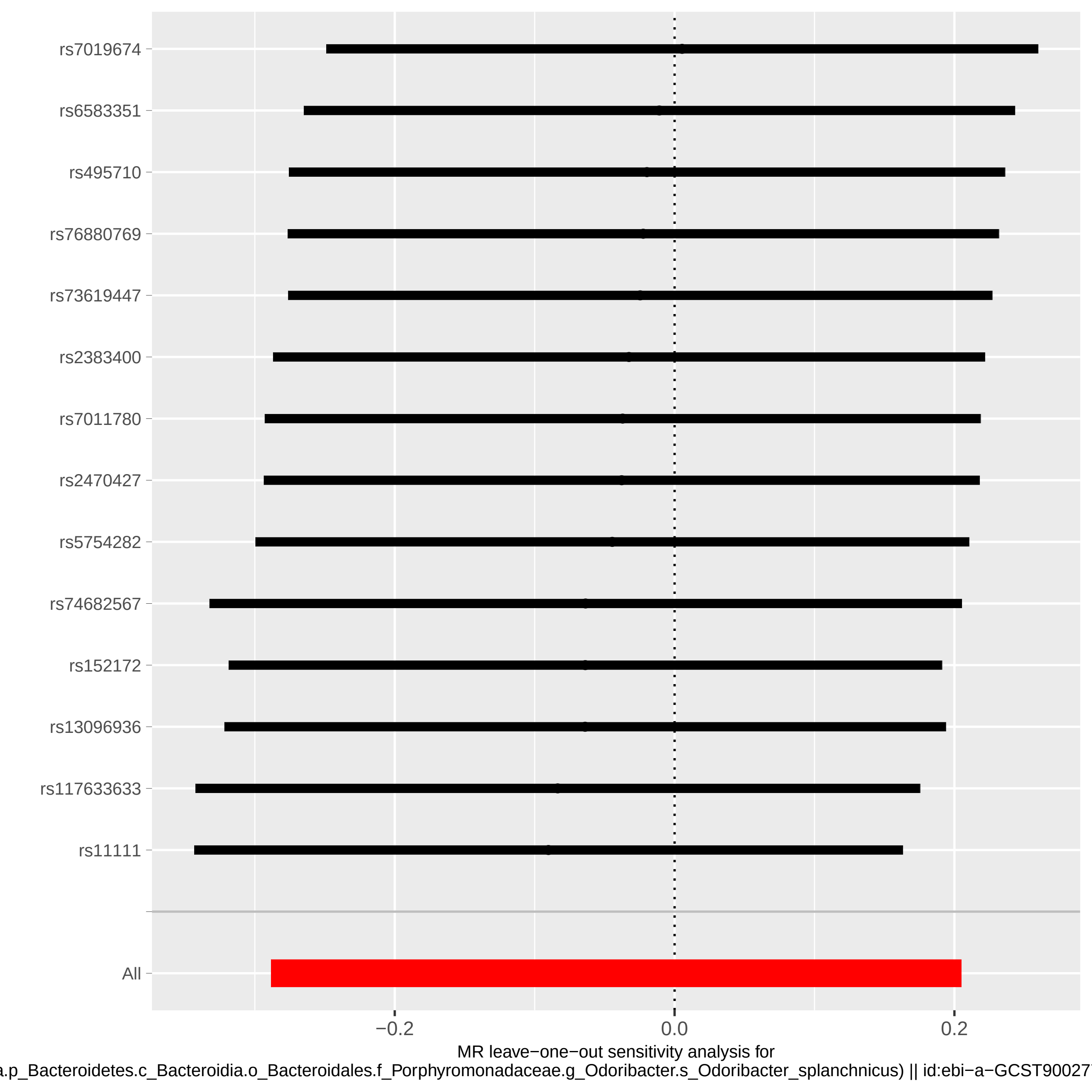

rs7019674
rs6583351
rs495710
rs76880769
rs73619447
rs2383400
rs7011780
rs2470427
rs5754282
rs74682567
rs152172
rs13096936
rs117633633
rs11111
All
−0.2
0.0
0.2
MR leave−one−out sensitivity analysis for
a.p_Bacteroidetes.c_Bacteroidia.o_Bacteroidales.f_Porphyromonadaceae.g_Odoribacter.s_Odoribacter_splanchnicus) || id:ebi−a−GCST90027
